# Gene expression in the brain of a migratory songbird during breeding and migration

**DOI:** 10.1186/s40462-016-0069-6

**Published:** 2016-02-15

**Authors:** John Boss, Miriam Liedvogel, Max Lundberg, Peter Olsson, Nils Reischke, Sara Naurin, Susanne Åkesson, Dennis Hasselquist, Anthony Wright, Mats Grahn, Staffan Bensch

**Affiliations:** Karolinska Institute, Department of Laboratory Medicine, Clinical Research Center, Karolinska University Hospital, SE-14186 Huddinge, Sweden; School of Natural Sciences, Technology and Environmental Studies, Södertörn University, SE-141 89 Huddinge, Sweden; Department of Biology, Molecular Ecology and Evolution Laboratory, Lund University, Ecology Building, SE-22362 Lund, Sweden; Centre of Environmental and Climate Research, Lund University, Ecology Building, SE-223 62 Lund, Sweden; Department of Biology, Centre for Animal Movement Research, Lund University, Ecology Building, SE-22362 Lund, Sweden; Max Planck Institute for Evolutionary Biology, AG Behavioural Genomics, August-Thienemann-Straße 2, 24306 Plön, Germany

**Keywords:** Migration, Microarray, Birds, Gene expression, Behavior, Calcium transport, Synapse, Phylloscopus trochilus

## Abstract

**Background:**

We still have limited knowledge about the underlying genetic mechanisms that enable migrating species of birds to navigate the globe. Here we make an attempt to get insight into the genetic architecture controlling this complex innate behaviour. We contrast the gene expression profiles of two closely related songbird subspecies with divergent migratory phenotypes. In addition to comparing differences in migratory strategy we include a temporal component and contrast patterns between breeding adults and autumn migrating juvenile birds of both subspecies. The two willow warbler subspecies, *Phylloscopus trochilus trochilus* and *P. t. acredula*, are remarkably similar both in phenotype and genotype and have a narrow contact zone in central Scandinavia. Here we used a microarray gene chip representing 23,136 expressed sequence tags (ESTs) from the zebra finch *Taeniopygia guttata* to identify mRNA level differences in willow warbler brain tissue in relation to subspecies and season.

**Results:**

Out of the 22,109 EST probe sets that remained after filtering poorly binding probes, we found 11,898 (51.8 %) probe sets that could be reliably and uniquely matched to a total of 6,758 orthologous zebra finch genes. The two subspecies showed very similar levels of gene expression with less than 0.1 % of the probe sets being significantly differentially expressed. In contrast, 3,045 (13.8 %) probe sets were found to be differently regulated between samples collected from breeding adults and autumn migrating juvenile birds. The genes found to be differentially expressed between seasons appeared to be enriched for functional roles in neuronal firing and neuronal synapse formation.

**Conclusions:**

Our results show that only few genes are differentially expressed between the subspecies. This suggests that the different migration strategies of the subspecies might be governed by few genes, or that the expression patterns of those genes are time-structured or tissue-specific in ways, which our approach fails to uncover. Our findings will be useful in the planning of new experiments designed to unravel the genes involved in the migratory program of birds.

**Electronic supplementary material:**

The online version of this article (doi:10.1186/s40462-016-0069-6) contains supplementary material, which is available to authorized users.

## Background

Bird migration is a complex behavior that involves a suite of traits evolved to optimize the movements between areas for reproduction, staging and winter survival [1]. Therefore, the combined behavioral, morphological and physiological traits associated with flight and navigation are characterized as migration phenotypes [2]. Comparative analyses of migratory and sedentary populations within passerine bird species, or between closely related species that differ in their migratory behaviors, have demonstrated strikingly parallel patterns of adaptive evolution on the phenotype level [3, 4]. The coordination of a set of complex behavioral adaptations has been referred to as ‘behavioral syndrome’ [5], a concept that has been used to discuss the evolution of migration [6, 7].

Quantitative genetic approaches have demonstrated heritable variation and strong selection for several key components of the migratory phenotype, such as migratory propensity, timing, distance and migratory orientation in birds [8, 9], migratory propensity in fish [10] and wing morphology in insects [11] and birds [12, 13]. In many species of long distance migratory songbirds, the core components of migration (timing, distance and direction) likely have a genetic basis since juveniles typically migrate on their own without influence of experienced adults [1]. Hence, we may expect differentially migrating bird populations to differ in genes encoding for several adaptations directly or indirectly linked to modulation of the migratory phenotype.

Whereas quantitative genetic studies demonstrate the presence or absence of within-species genetic variation associated with migratory traits, they do not provide data required for testing whether the same or different genes are involved in the migratory phenotypes of different species [14]. The work of identifying the genes that influence the behavioral adaptations associated with migration is still in its infancy and among the so far proposed candidates there is none that has consistently shown to be influencing migratory traits across species. The most extensive study comparing differential expression patterns between phenotypes used microarray analyses of monarch butterflies *Danaus plexippus* to compare summer and migratory phenotypes. This study identified 40 differentially expressed genes between seasons using brain tissue [15]. Two genes of particular interest were the clock gene *vrille* and the locomotion-relevant TβH (*tyramine beta hydroxylase*) gene as their presumed functions directly link to migratory components (timing and movement respectively). A recent genome re-sequencing study of monarch butterflies identified a gene (collagen IV) influencing flight muscle functioning with the migratory alleles resulting in improved flight efficiency [16]. One study on European blackcaps *Sylvia atricapilla* [17] analyzed six genes with functions suggesting potential association to the migratory program. A microsatellite polymorphism in the 3′-UTR of the gene encoding Adenylate Cyclase Activating Polypeptide 1 (*ADCYAP1*) was found to explain a small but significant proportion of migratory restlessness within populations, as well as variation in migratory distance across populations [17].

In Northern Europe, two geographically separated subspecies of the willow warbler (*Phylloscopus trochilus*) have been used extensively to study differential migration behavior with regard to migration direction and wintering locations in Africa [18–20]. The subspecies *P. t. trochilus,* breeding in southern Scandinavia, Britain and western parts of Central Europe, migrates towards the south-west in autumn to wintering grounds in West Africa. In contrast, the subspecies *P. t. acredula,* breeds in northern Scandinavia, Finland and Russia, migrates south-south-east to eastern and southern Africa [18–21]. Whereas the contact zone between the two species east of the Baltic Sea is complex, with different positions of cline centres and cline widths for size, coloration and migration strategy, they form a well-defined and 200 km wide contact zone in central Scandinavia [21, 22]. The subspecies are inseparable with regard to neutral genetic markers (e.g., mitochondrial DNA, microsatellites and AFLPs; [21]) and breeding habitat [23] suggesting that the few divergent genomic regions are maintained by selection associated with their different migration phenotypes [24].

To identify molecular components of the brain that are potentially involved in migration direction behaviour, we used a microarray chip designed from expressed sequence tags (ESTs) in brain tissue of zebra finches [25] to compare genome-wide expression of mRNA transcripts in brain tissue collected from two willow warbler subspecies with different migratory strategies during two crucial life history stages, i.e. breeding in adults as well as the early phase of migration in juvenile birds. Our rational behind choosing the brain as the target for the analyses is because it is the tissue where migration-related decision-making gets integrated. We decided to use the whole brains rather than separate regions because we are lacking precise information and characterisation of focal brain regions that process migration-related decision making genes. Based on the low degree of genetic differentiation between the subspecies [21, 24] and the different challenges the birds are facing during breeding and migration, we expect to find more difference in gene expression when comparing breeding and autumn samples within the subspecies than when comparing the subspecies within seasons. If genes involved in migration-direction decision-making are included in our microarray chip, we expected these to be differentially expressed when comparing the autumn samples of the two subspecies. However, given the lack of knowledge as to whether expression of such genes is restricted to the migration period, we included the overall contrast between the subspecies (within seasons) in our analysis.

## Methods

### Fieldwork and sample collection

**Fig. 1:**
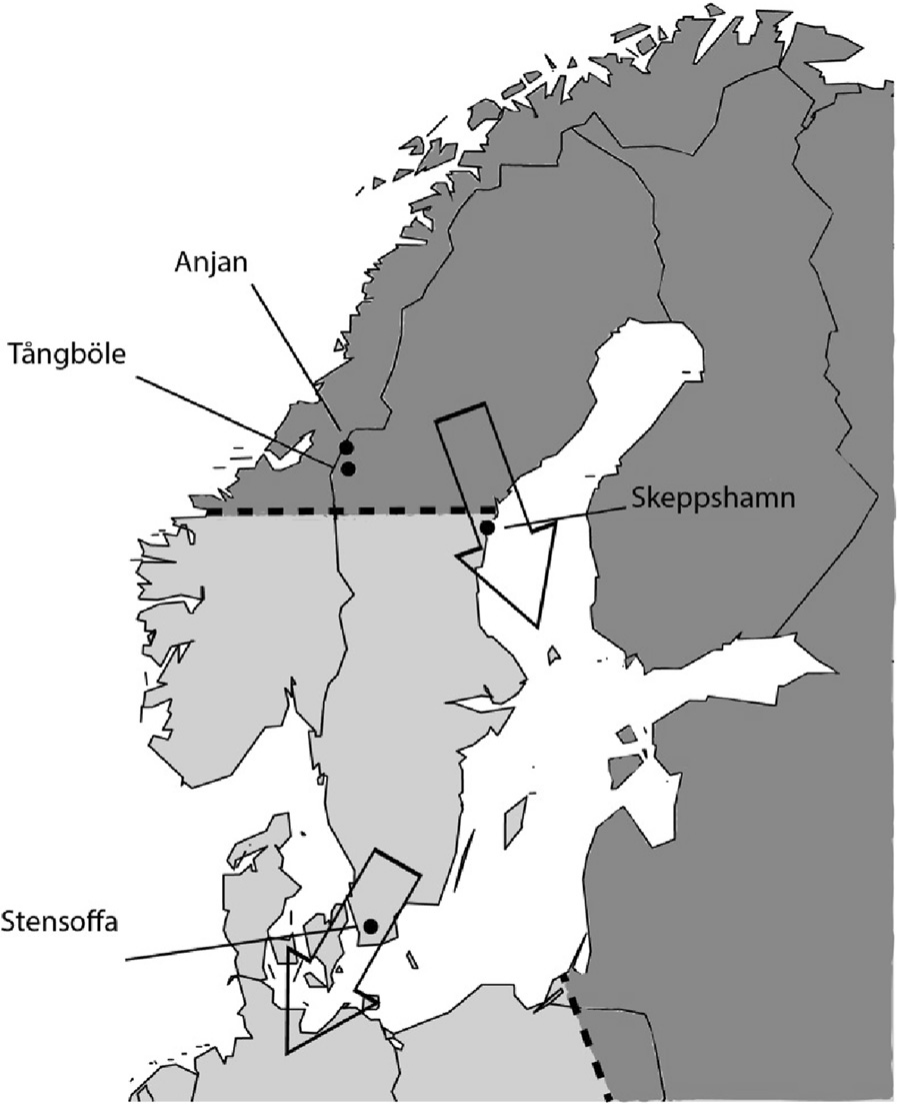
Locations of willow warbler sampling sites in Sweden. The stippled lines refer to the approximate positions of the centres of two migratory divides around the Baltic sea, separating the ranges of SW migrating *P. t. trochilus* (light grey) and SE migrating *P. t. acredula* (dark grey). Breeding birds were captured at Stensoffa, Tångböle and Anjan, autumn birds at Stensoffa and Skeppshamn. The autumn capture site for *P. t. acredula* at Skeppshamn is south of the centre of the hybrid zone. At the date for capture at Skeppshamn (19 August), birds are expected to origin from further north in Scandinavia (well within the range of *P. t. acredula*) whereas locally hatched birds should already be further south on their move to the wintering grounds

We collected 32 birds in 2008, eight from each subspecies and season, of which 26 could be used for the final analyses of the microarray data (see below). Second calendar year or older (2 cy+) willow warbler males defending breeding territories were captured in mist nets with the help of song playback at one site in southern Sweden (8 male *P. t. trochilus* collected, Krankesjön, 55°43′N, 13°25′E, 8–19 May 2008) and at two sites in northern Sweden (4 males, Tångböle, 63°22′N, 12°36′E, 30 May 2008; 4 males, Anjan, 63°43′N, 12°32′E, 31 May 2008, *P. t. acredula*) (Fig. 1). At the early phase of migration we opportunistically captured birds in mist nets placed in habitats preferred by willow warblers at one site in southern Sweden (8 birds collected, Krankesjön, 15–16 August 2008) and one site in northern Sweden (8 birds collected, Skeppshamn, 62°23′N, 17°44′E, 19 August 2008). We chose to sample inexperienced juvenile birds on their first migration because they typically account for >95 % of willow warblers captured in mist-nets during autumn migration, and importantly, their migratory programme is purely inherited and does not yet include a learning component. The autumn capture site for *P. t. acredula* is slightly south of the centre of the hybrid zone (Fig. 1). At this date (19 August) the captured birds are expected to origin from further north in Scandinavia (well within the range of *P. t. acredula*), whereas locally hatched birds should already be further south on their move to the wintering grounds. At each site, we captured more birds than we collected for the microarray experiment. We selected the birds for collection based on the following criteria: (i) 2 cy + males should have been netted within 1 min after starting tape recorder play-back of song, (ii) tissues collected within 5 min after the bird was removed from the net, (iii) 1 cy males should have completed their post-juvenile moult, i.e. being at least 60 days old [26] and (iv) carrying at least a moderate fat load (score ≥ 3; [27]). All birds were collected between 6–12 am. Birds were sacrificed by decapitation and the entire brain was immediately transferred into a tube containing RNAlater™ RNA stabilization Reagent (Qiagen). Samples were kept in the field for up to one week and then at −80 °C prior to RNA extraction.

### RNA preparation and analysis

Nine months post collection of the samples (May and June 2009) brains were removed from the RNAlater buffer and homogenized in 1 ml QIA-zol Lysis Reagent per 100 mg tissue (Qiagen) using a TissueLyser (Qiagen). The samples were processed in batches of four individuals (one from each group) using the RNeasy Lipid Tissue Mini Kit (Qiagen) according to the manufacturer’s instructions. Samples were quality-controlled by electrophoresis on formaldehyde agarose gels and confirmed to have an acceptable 260 nm/280 nm absorption ratio (all values between 1.95 and 2.05) using an Ultraspec 3000 spectrophotometer (Pharmacia).

For this project, we had 29 Lund-zf2 microarray chips available and therefore RNA samples with the highest RNA Integrity Numbers [28] were selected (8 samples from each group except for *P. t. acredula* in autumn for which only 5 samples passed the RNA quality criteria). The Lund-zf2 array is a custom Affymetrix array with probe sets representing 23,136 zebra finch ESTs expressed in brain tissue. Each EST probe set is represented by eleven 25 bases long oligonucleotides (probes) and together the probe sets represent about 15,800 non-redundant gene models, for details see [25]. The microarrays were hybridized and analyzed at the Swegene Center for Integrative Biology at Lund University (SCIBLU genomics, http://www.lth.se/sciblu) using standard procedures (for details, see [29]). Across-species array hybridization is challenging as it may result in measured expression differences that are methodological artefacts that needs to be controlled for. Sequence mismatches between the probe and the target may for example underestimate the expression level. We have taken several measures to minimize such biases: First, all probes that had failed to hybridize against genomic DNA from willow warblers were removed [25], see details below. Second, this microarray chip has previously been thoroughly evaluated for across-species hybridization in the common whitethroat *Sylvia communis* [28–30], a species positioned at the same phylogenetic distance to the zebra finch as the willow warbler. In these studies, the patterns of sex-biased gene expression were very similar for the zebra finch (within-species array hybridization) and the whitethroat (across-species array hybridization), both regarding the specific genes found being differentially regulated between the sexes and the magnitude of sex-biased expression.

### Data analysis

All the birds were sexed by standard molecular methods [31] and the collected wings of the birds captured during the autumn were carefully checked to confirm that they had been correctly aged [32]. Of the 29 analyzed samples (*P. t. trochilus*, 8 summer and 8 autumn; *P. t. acredula*, 5 summer and 8 autumn) we excluded two samples from the autumn birds (one from each subspecies) that were subsequently shown not to be juvenile males (one juvenile female and one adult male). All statistical analyses of the microarray dataset were carried out using R version 2.15.1 (R Core Development Team 2012). Quality of the array results was evaluated using the affyPLM package. A background correction for each probe was performed using the robust multi-array average (RMA) method as implemented in the affy package. Subsequently, the probe signals were normalized using RMA normalization. We evaluated the normalizations by inspecting the MA, NUSE and RLE plots before and after RMA normalization. The expression data was further evaluated by hierarchical clustering (average clustering method using Euclidean distances), and principal component analysis to identify possible outlier arrays (data not shown). One array (from a breeding *acredula* male) was marked as an outlier and was therefore removed from the subsequent analyses. Data from the remaining 26 arrays was re-normalized followed by a new round of quality control. Consequently, our reported analyses were carried out on data from eight breeding *P.t. trochilus* males, four breeding *P. t. acredula* males, seven migrating *P. t. trochilus* males, and seven migrating *P. t. acredula* males.

Since low probe hybridization signals can result not only from a low gene expression level but also from a poor sequence match of willow warbler transcripts to the probes designed from zebra finch ESTs, we made use of a previous genomic DNA hybridization data using the Lund-zf array [25] to determine cut-off parameters. This experiment contained data for two arrays each of which had been hybridized with pooled DNA from eight *P. t. trochilus* and eight *P. t. acredula* birds, respectively. Probes that had a signal at least two standard deviations above the mean background expression level on at least one of these arrays were flagged as present (i.e., the willow warbler sequence matches the zebra finch probe), while probes yielding signals below two standard deviations were flagged as absent (i.e. poor cross-species sequence match). Out of the 23,139 probe sets on the zf-2 array, 1,030 (4.4 %) had less than eight functional probes (Affymetrix standard cut-off; [25]) after subtraction of poorly matching probes and were excluded from the downstream analyses (Additional file 1: Figure S1). For the remaining 22,109 probe sets, confirmed to show significant hybridization signals to willow warbler DNA, we calculated the mean intensities for probes in each probe set as estimates of gene expression levels.

Probe sets were linked to genes using blastn 2.2.24+ in the toolset of NCBIs standalone blast (http://blast.ncbi.nlm.nih.gov/Blast.cgi?CMD=Web&PAGE_TYPE=BlastNews#1). The database (zebra finch gene set version 3.2.4) was downloaded from Ensembl (www.ensembl.org) using the biomart tool and included 18,581 annotated genes together with potentially uncharacterized untranslated region 1,000 bp up- and downstream of the first and last exons, respectively. Flanking sequences were included to allow identification of EST sequences corresponding to untranslated regions of gene transcripts. These blast results were then evaluated using an in-house Python script (supplementary file) in order to remove probes with more than one possible hit (second hit within an e-value range of 10^3^ of the first) and alignments shorter than 50 bp. The four sample groups were compared in order to identify gene expression differences associated with the effects of season and subspecies. Two additional comparisons were made by combining sample groups according to subspecies or season. To avoid artefacts associated with identification of false positives due to multiple testing (i.e. erroneously rejecting the null hypothesis of no difference between groups) we used the false discovery rate correction (FDR) with a statistical cut-off value of 5 % [33].

Significant enrichment of differentially regulated genes associated with gene ontology terms (GO terms) was determined using the CORNA R package [34]. We selected the annotated and significantly differentially regulated genes between breading and migratory season (1,640) for gene ontology analysis. We did not carry out GO term analyses with respect to subspecies/migratory strategy as the numbers for significantly differentially regulated genes between subspecies were too low for meaningful subsequent analyses. Gene descriptions were gathered from Ensembl and statistical significance of the enrichment was evaluated using a Fisher’s exact test with correction for false discovery rate (FDR). The inbound relationships between GO terms were examined using the web tool Revigo (http://revigo.irb.hr/) and the method SimRel [35]. SimRel calculates a differentiation score, pairing all GO terms against each other. Revigo was used to create scatterplots of the physiological differences between GO terms of biological processes below *p* < 0.05.

## Results

**Fig. 2:**
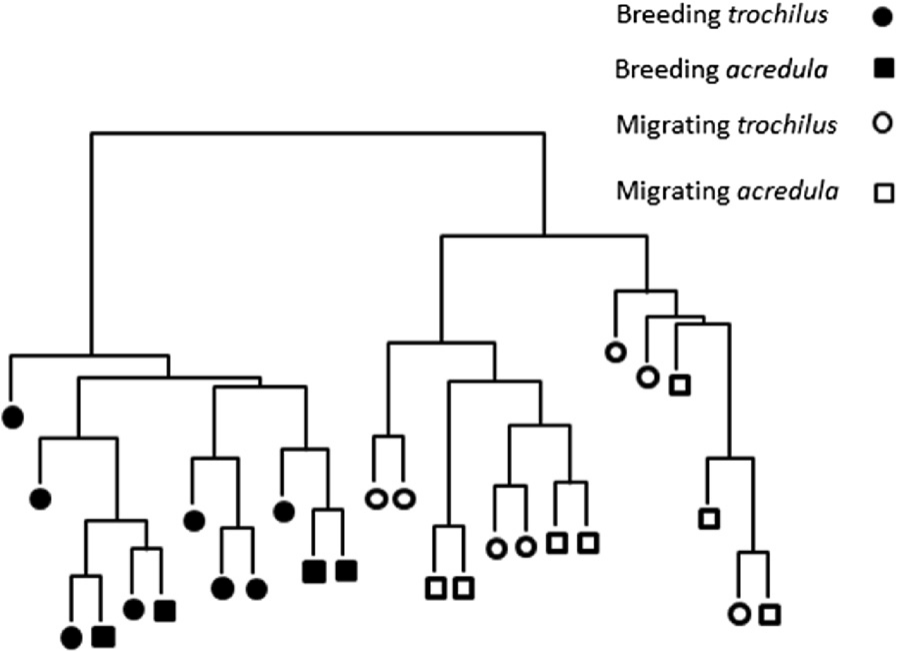
Hierarchical clustering of the array expression profiles for the 26 male willow warblers included in the analyses. Note that the breeding birds were adult and the migrating birds were juveniles

We successfully matched 11,898 EST probe sets (51.8 % of the original 22,109 represented on the microarray) to a total of 6,758 annotated zebra finch genes. A cluster dendrogram was created from the probe sets to determine how overall gene expression patterns differ between the willow warbler samples and to describe their hierarchical relationships (Fig. 2, Additional file 1: Figure S2). Gene expression differences associated with seasonal sampling time (summer/ adult breeding versus autumn/juvenile migration) dominated the clustering result and allowed to perfectly separate the birds. Within both seasonal clusters, there was no clear grouping of the samples according to overall RNA patterns associated with the different subspecies (i.e. migratory strategy). We conclude that the main difference in RNA levels between the samples was found between willow warblers collected during different seasons and that RNA expression differences distinguishing subspecies were substantially fewer.

### Estimated number of differentially expressed genes

**Fig. 3:**
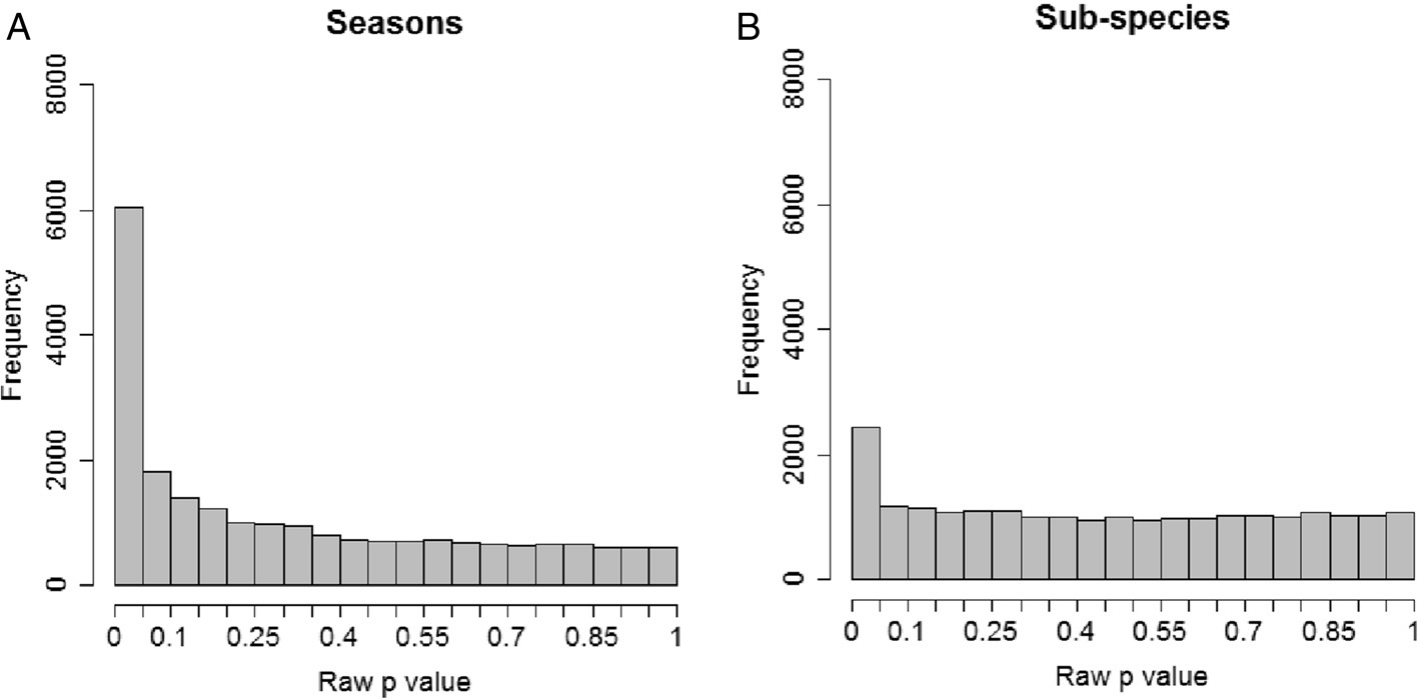
Distribution of raw p values between seasons (**a**) and between subspecies (**b**) of willow warblers. Note that the season contrast is between adult breeding and juvenile migrating birds

In order to estimate the approximate number of genes that are differently expressed in birds from different seasons (breeding adults versus migrating juveniles) and between different subspecies, we plotted the distribution of raw p-values (testing the significance of gene expression differences) for the 22,109 probe-sets (Fig. 3). Both seasonal and subspecies comparisons had *p*-value distributions that were highly skewed towards low values. Between summer and autumn, there were 6,054 probe sets with *p*-values < 0.05 compared to an expected number of 1,105 (5 % of all probes) for a random distribution. For the subspecies comparison, 2,430 probe sets had a *p*-value < 0.05, fewer than for genes differing between seasons, but more than twice as many as expected by chance. Although that this suggests that a substantial number of genes were differentially expressed in both comparisons, the lowest 5^th^ percentile are inflated by −20 % and −50 % false positives, respectively.

### Gene expression differences between breeding adults and juvenile migrants

**Fig. 4:**
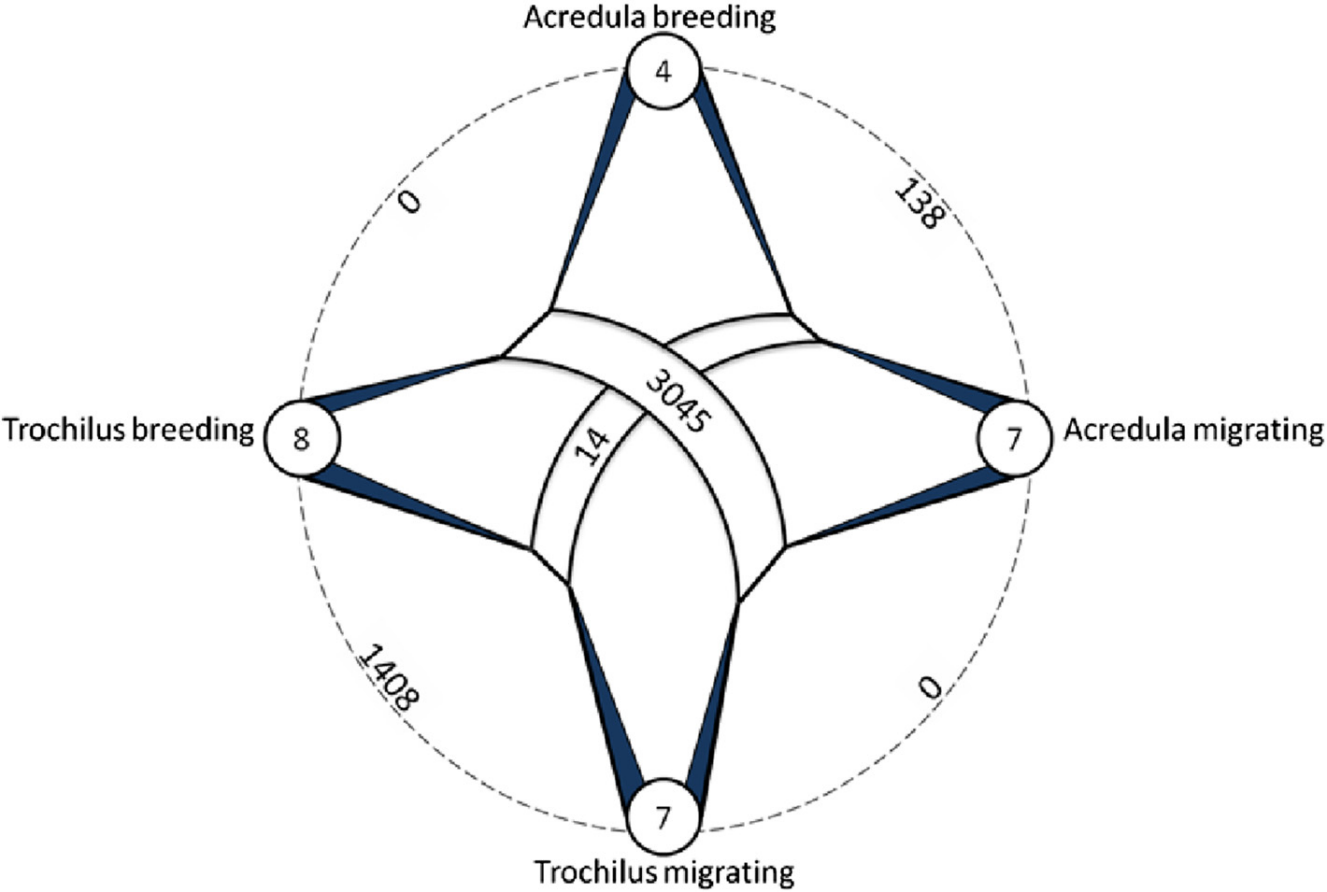
Number of significantly regulated genes (out of 22109 examined) in whole brain RNA extracts between adult breeding and juvenile migrating willow warblers of the subspecies *P. trochilus trochilus* and *P. t. acredula*. Numbers inside circles show the number of birds from each subspecies and season. The outer lines with associated numbers show the comparisons made between samples and the number of differentially expressed genes (adjusted *p* value < 0.05), the middle bands represent combined sample comparisons

Within subspecies, the comparison between breeding adults and juvenile migrants on their first migratory journey identified 138 probe sets that were differently expressed (<5 % FDR) in *P. t. acredula* and 1,408 probe sets in *P. t. trochilus* (<5 % FDR) (Fig. 4). An overlap of 73 probe sets was differentially expressed in both subspecies. The lower number of differentially expressed genes identified for *P. t. acredula* probably reflects the lower statistical power associated with the smaller number of breeding birds analyzed for this subspecies (*n* = 4 in *acredula* vs. *n* = 8 in *trochilus*).

Because the effect of subspecies was small in the overall clustering of the samples (Fig. 2), we combined the data from both subspecies for each season to increase the statistical power of the comparison between seasons. In this combined analysis, we identified 3,045 probe sets that were differentially expressed between seasons (FDR adjusted *p*-value <0.05, 13.8 % of the total). Among the top 20 significant and annotated genes (Table 1) there are several genes associated with neurone functions such as Neurexin 1 beta involved in synapse formation [36], as well as Parvalbumin genes, which encode markers identifying a subclass of interneurons [37]. Among identified genes not shown in Table 1 (Additional file 1: Table S1) were the two clock genes NAPS2 (FDR 0.006) and ADCYAP1 (FDR 0.002), of which ADCYAP1 has previously been reported to associate with migration tendency and migratory restlessness in blackcaps [17]. ADCYAP1 had 1.2-fold higher RNA levels in breeding compared to migrating willow warblers. The overall the proportion of up- and down-regulated genes between seasons was similar with about half (50.9 %) of the 3,045 differentially expressed probe sets showing higher RNA levels in autumn than in summer. However, the most differentially expressed genes tended to be more frequently up-regulated in autumn than in summer. This is shown by the percent of autumn up-regulated genes calculated for successive blocks of 100 genes (Additional file 1: Table S1); 77 % among the first block (1–100), 69 % (101–200), 62 % (201–300, 59 % (301–400) and 54 % (401–500).

**Table 1:** Top list of the 20 annotated genes that differed most significantly between samples from breeding adult and juvenile migrating male willow warblers

Ensembl gene ID	Gene name	Fold change	FDR	Description
ENSTGUG00000013386	ATP1A1	2.00	8.92E-09	ATPase, Na+/K+ transporting, alpha 1 polypeptide
ENSTGUG00000006383	NRSN1	−1.35	8.92E-09	neurensin 1
ENSTGUG00000010974	HIVEP2	1.61	8.92E-09	human immunodeficiency virus type I enhancer binding protein 2
ENSTGUG00000007668	FSCN1	1.51	1.11E-08	fascin actin-bundling protein 1
ENSTGUG00000001697	ARPP21	1.61	2.08E-07	cAMP-regulated phosphoprotein, 21 kDa
ENSTGUG00000005731	NRXN1	1.33	3.97E-07	neurexin 1
ENSTGUG00000013106	FAM49A	1.43	6.01E-07	family with sequence similarity 49, member A
ENSTGUG00000000314	CREBRF	−1.74	6.65E-07	CREB3 regulatory factor
ENSTGUG00000005128	KIAA922	1.36	7.20E-07	KIAA0922
ENSTGUG00000009005	TEKT1	1.87	1.06E-06	tektin 1
ENSTGUG00000001522	COL1A2	2.32	1.42E-06	collagen, type I, alpha 2
ENSTGUG00000003383	BRINP3	1.37	1.98E-06	bone morphogenetic protein/retinoic acid inducible neural-specific 3
ENSTGUG00000002940	SRPK2	1.17	2.04E-06	SRSF protein kinase 2
ENSTGUG00000002420	C8G	−1.61	2.04E-06	complement component 8, gamma polypeptide
ENSTGUG00000011663	PMP2	3.98	2.20E-06	peripheral myelin protein 2
ENSTGUG00000006231	NHLRC1	1.51	2.41E-06	NHL repeat containing E3 ubiquitin protein ligase 1
ENSTGUG00000012725	DCUN1D5	1.20	3.28E-06	DCN1, defective in cullin neddylation 1, domain containing 5
ENSTGUG00000003264	TLE4	1.27	3.80E-06	transducin-like enhancer of split 4
ENSTGUG00000012354	PLEKHA5	1.30	4.23E-06	pleckstrin homology domain containing, family A member 5
ENSTGUG00000010713	PVALB-2	−1.29	4.38E-06	Taeniopygia guttata parvalbumin-like

Gene name and descriptions are taken from the Ensembl database, probe sets without annotations were excluded. Positive fold change values indicate the gene is more expressed in migrating juveniles than in breeding adults, and vice versa for negative values. The *p*-values are corrected for multiple testing using the Benjamini & Hochberg (FDR) correction

### Gene expression differences between subspecies

The overall clustering of the expression profiles (Fig. 2) showed that the difference in the number of differentially expressed genes between subspecies was small. In line with this, within-season comparisons of gene expression differences between the subspecies did not provide support for any differences between subspecies either in summer or in autumn (Fig. 4). In order to increase the power of the comparison, we compared probe data for all birds from each subspecies regardless of season. Expression data from 11 *P. t. acredula* birds were thus compared to data from 15 *P. t. trochilus* individuals. This comparison identified 14 differentially expressed probe sets (FDR adjusted *p*-value <0.05, 0.6 % of the total) (Fig. 4). Including the interaction term subspecies x season only marginally changed the results (12 differentially expressed probe sets of which 10 overlapped with the 14 detected without the interaction). Seven of the 14 significant genes did not have Ensembl annotations or were annotated as uncharacterized. Nonetheless, a significant and annotated gene of potential interest is the PPP3CA gene, which encodes a calcium-regulated (via Calmodulin) protein phosphatase. PPP3CA is the only gene that was shown to be significantly changed in comparisons of both the seasons and subspecies, perhaps due to a role of calcium-related signalling in both contexts. PPP3CA mRNA levels were significantly higher in migrating birds compared to breeding birds, and higher in the southern subspecies, *P. t. trochilus,* compared to the northern subspecies, *P. t. acredula*.

### Differentially regulated genes are enriched in specific functional gene classes

We then performed a gene ontology analysis, in order to determine whether genes differentially regulated between adult breeding and juvenile migrating birds were significantly enriched within particular categories of biological functions. The 1,640 differentially regulated and annotated genes between adult breeding and juvenile migrating birds were significantly enriched for “positive regulation of gene expression” (GO:0010628) and “calcium ion transport” (GO:0006816) after correction for multiple testing (Table 2). Thirteen of the significantly regulated annotated genes in the category “calcium ion transport” were directly involved in the formation and function of calcium channels (Table 3). Out of the 13 genes, 10 were up-regulated during migration (Table 3).

**Table 2:** Lists of the ten most significant results from the gene ontology enrichment analyses between breeding adult and juvenile migrating willow warblers

GO description	Adj *P*-value	Total	Observed (Expected)
positive regulation of gene expression	0.0084	40	13 (3)
calcium ion transport	0.04	30	10 (2)
translation	0.15	117	21 (9)
calcium ion transmembrane transport	0.19	20	7 (2)
structural constituent of ribosome	0.28	95	17 (7)
ribosome	0.28	98	17 (8)
RNA polymerase II regulatory region sequence-specific DNA binding	0.28	4	3 (0)
protein serine/threonine kinase activity	0.28	159	3 (12)
protein domain specific binding	0.28	41	9 (3)
regulation of mRNA stability	0.28	5	3 (0)

.*P*-values are calculated from Fisher’s exact test and adjusted using FDR. ‘Total’ refer to all the genes included in that gene ontology term and ‘Observed’ is the number of genes found

**Table 3:** Expression differences of genes included in the ontology term referred to as “calcium ion transport”

Gene name	Fold change	Description
ATP2B2	1.35	ATP2B2 ATPase, Ca++ transporting, plasma membrane 2
ATP2B1	1.20	ATPase, Ca++ transporting, plasma membrane 1
TRPM7	1.19	Transient receptor potential cation channel, subfamily M, member 7
SCN3A	1.23	Sodium channel, voltage-dependent, type III, alpha subunit
CACNA1B	1.32	Calcium channel, voltage-dependent, N type, alpha-1B subunit
ITPR1	1.24	Inositol 1,4,5-triphosphate receptor, type 1
PPP3CA	1.40	Protein phosphatase 3, catalytic subunit, alpha isozyme
CACNG2	−1.17	Calcium channel, voltage-dependent, gamma-2 subunit
GRIN1	−1.22	Glutamate receptor, ionotropic, N-methyl D-aspartate 1
ATP2C1	1.16	ATPase, Ca++ transporting, type 2C member 1
CACNG3	1.24	Calcium channel, voltage-dependent, gamma-3 subunit
TRPC5	1.34	Transient receptor potential cation channel, subfamily C, member 5
ANXA6	−1.30	Annexin A6

Fold change differences are between migrating juvenile and breeding adult willow warbler males, with positive values being more expressed in the brain tissue samples of migrating juveniles

## Discussion

The two closely related willow warbler subspecies with clearly different migratory strategy provide a good system to study changes in gene expression associated with migration. As expected, the overall gene expression differed substantially between breeding adults and migrating juveniles, whereas the differences between the subspecies were much smaller. This is in line with the general assumption that differences in the migratory phenotype, such as migratory direction, are likely governed by a limited number of genes. This might also be due to the fact that the expressions of the genes involved in these processes are time-structured or tissue-specific in ways which our approach failed to uncover. The low level of differential gene expression between the subspecies is in line with previous studies examining sequence-based differences [21, 38]. In these analyses, the subspecies were inseparable based on mitochondrial DNA, microsatellites and at >99 % of 500 investigated AFLP markers. Analyzes of sequence differences in the transcriptome [24] has confirmed the main absence of genomic divergence between the two subspecies of willow warblers, with the exception of three regions (each of a few Mbp) on the putative chromosomes 1, 3 and 5. However as this data was based on pooled RNA samples in combination with a normalization procedure to equalize transcripts across the transcriptome [24], we could not use it for evaluating expression differences between the subspecies.

### Large gene expression differences between breeding adults and migrating juveniles

The highly skewed raw *p*-value distribution (Fig. 3a) suggested that the actual number of genes involved in seasonal or age dependent differences of expression is higher than those 3,045 determined using the Benjamini and Hochberg false discovery rate [33]. Our data suggest that as many as 22 % of EST probe sets (4,953 out of 22,109) may be differentially expressed between season or age classes. To correctly set the tradeoff between Type I and Type II errors is a well-known problem in all gene expression studies [39] and it has been suggested that well under 50 % of truly differently expressed genes fail detection even in well-powered studies [40]. Furthermore, this number could also be an underestimation because of the experimental setup where the whole brain was investigated. Differences in gene expression restricted to small regions of the brain might be swamped by mRNA levels from larger brain regions, or possibly due to upregulation in one region and down-regulation in others [41].

Many of the gene expression differences seen in this study are probably due to the physiological requirements of different seasons and associated life history stages, but still there are alternative explanations that cannot be ruled out. For example, our sampling protocol does not allow discriminating between season and age differences of the population. The analyzed migrating male willow warblers were all in their first year (2–3 months old), while the breeding individuals consisted of males that were one year or older. Thus, any putative age-dependent difference shaping the pattern will be picked up as season differences in our experimental design.

Inspection of the 3,045 probe sets with the lowest *p*-values (Table 1) reveals a high number of neuron and calcium flux related genes. Regulation of calcium concentrations have been linked to multiple functions within brain cells such as neuronal transmission, excitatory synapses, cell growth, proliferation and synaptic plasticity [42]. One of the significantly differentiating expressed gene, the adenylate cyclase activation polypeptide 1 (ADCYAP1, Additional file 1: Table S1), has previously been associated with migration behavior. It has been shown that the length of the 3′ untranslated region (UTR) on ADCYAP1 alleles partly predicts migratory restlessness within some bird species [17, 43]. Because we find ADCYAP1 mRNA levels to be lower during migration, our study suggests that ADCYAP1 expression might inhibit migratory restlessness. But since the breeding birds were adults and the migrating birds were juveniles on their first migration we cannot disentangle the effects of season from any putatively confounding age effect. The gene ADCYAP1 is highly conserved among birds and mammals, and encodes the protein PACAP that together with its selective receptor PAC1 is integral to anxiety behavior and stress responses in the limbic system of the brain [44].

The gene ontology enrichment analysis identified the terms “positive regulation of gene expression” and “calcium ion transport” as up-regulated in juvenile migrants compared to adult breeding willow warblers. The up-regulation of genes that increase gene expression agrees with our observed pattern the most differentially expressed genes being up-regulated in migrating juveniles compared to breeding adults.

Our background set included all genes that we could identify as matching the array probes. Since the sample list contains predominantly brain-expressed genes while our background set contains both brain expressed and non-brain expressed genes, one concern might be that typical nervous-tissue related genes become significance just because our samples consisted of brain tissue. However, we only found one of the possible brain-specific GO categories (calcium ion transport) as being significantly enriched; this would not be expected if we were just generally finding brain GO classes. Our data do not allow us to say why calcium ion transport is up-regulated in the brain during migration, but one potential explanation could involve the proliferation of neurons and creation of new synaptic connections needed during migration. Migration flights require the ability to use biological compasses and maps for navigation [2, 45–47], which are expected to be associated with high neuronal activity and a capacity to integrate information from different sensory systems [48, 49]. Another possibility is that the biological processes differing between the two seasons are mainly linked to the breeding period. For example, earlier studies suggest that genes involved in ion transport are repressed during singing behavior [48].

### Small subspecies-dependent gene expression differences

The raw *p*-value distribution suggests that up to around 1,300 probe sets (6 %) might potentially involve genes differentially expressed in both subspecies, even those that cannot be individually confirmed with statistical confidence. Only 14 genes (0.06 %) were statistically supported (adjusted *p* < 0.05) to have differential expression between the two subspecies after adjusting for multiple testing. A study investigating subspecies differences between the hooded crow [49], *Corvus corone cornix* and the carrion crow, *Corvus corone corone*, that mainly differ in plumage color, found a much higher number of differentially expressed genes (106). Although no gene overlapped with this study, the cellular functions of neural regulation were differentially expressed in both studies.

The strongest supported probe set was similar to adenylate kinase isoenzyme 5 (AK5) which had a higher expression in *P. t. trochilus* than in *P. t. acredula* (FC 1.52). This protein is predicted to sense cellular energy levels [50]. Another significant gene is PPP3CA (higher expression in *P. t. acredula* than in *P. t. trochilus;* FC 1.90), which encodes the catalytic subunit of serine/threonine-protein phosphatase (alpha isozyme), a catalytic subunit of the phosphatase calcineurin. PPP3CA was the only gene with significant expression change in both the seasonal (autumn > summer) and subspecies (*P. t. trochilus* > *P. t. acredula*) comparisons.

### Differentially regulated genes involved in neuronal signaling

The experimental approach used for this first study on gene expression differences between two contrasting migratory strategies in willow warblers is limited by both methodological and conceptual factors, and is not sufficient for identifying all the specific genes involved. Our tentative list of genes differentially expressed in the brain between breeding adults and migrating juveniles as well as between subspecies indicate that specific functional areas and classes might be important for adaptation to migration and associated traits. However, more experiments are needed to confirm this as our seasonal classification criterion is confounded by age. With these limitations in mind, our study suggests an importance of genes involved in establishment of neuron networks (e.g., synapse formation) and neuron signaling (calcium ion transport). Both these functions might potentially play important roles involved in the cognitive/behavioral differences that characterize birds in different seasons, age classes and/or different subspecies. Calcium signaling is involved both in neuron firing and more long-term plasticity changes in neurons that fire repeatedly, leading to calcium-mediated signaling to the nucleus in cells in which calcium levels are elevated during longer times [51, 52]. Based on the present results, it will be possible to focus future studies on which calcium transporter subunits are differentially expressed in different brain regions. In such studies, markers for different classes of neurons should also be studied. Measurements of mRNA levels in different brain regions should also be complemented by *in situ* hybridization studies to determine whether the differentially expressed genes are predominantly expressed in neurons.

## Conclusion

Our study suggests that relatively few genes are involved in shaping the phenotypic differences in migratory strategies between the willow warbler subspecies. However, our snapshot approach (one tissue, summer and autumn) cannot rule out that major differences in gene expression exist in other tissues or at other time points. For example, gene expression differences restricted to small regions of the brain would likely not be detected in analyses of total brain mRNA extracts. Critical expression differences might also be timed to when the birds execute the directed flights, i.e. after sunset, whereas the samples we analyzed were collected in mornings. A tighter coupling of differential gene expression to migration direction choice will require development of experimental migration-related endpoints that can be measured in controlled experiments using birds in captivity where relevant cues (e.g. magnetic field, polarized light, direction of sun set) can be manipulated [53, 54]. Such studies should of course make use of the advancements in next generation sequencing technologies such as RNA-seq which will be more powerful to detect differences in gene expression than our microarray approach.

## Additional file

Additional file 1: The distribution of positive probes per probe set is shown in **Figure S1.** The hierarchical clustering including the discarded samples is shown in **Figure S2**. Gene IDs, fold-change differences and FDR *P*-values for each of the 22,109 probe sets are available in **Table S1**. Microarray data are available in the ArrayExpress database (www.ebi.ac.uk/arrayexpress) under accession number E-MTAB-4102. (ZIP 1810 kb)
