# Supplementary material for: Gene expression in the brain of a migratory songbird during breeding and migration
Source: Mov Ecol. 2016 Feb 15;4:4. doi: 10.1186/s40462-016-0069-6 (PMC4753645; doi:10.1186/s40462-016-0069-6)
Supplement: Additional file 1: — The distribution of positive probes per probe set is shown in Figure S1. The hierarchical clustering including the discarded samples is shown in Figure S2. Gene IDs, fold-change differences and FDR P-values for each of the 22,109 probe sets are available in Table S1. Microarray data are available in the ArrayExpress database (www.ebi.ac.uk/arrayexpress) under accession number E-MTAB-4102. (ZIP 1810 kb) [file 40462_2016_69_MOESM1_ESM.zip › Figure S1.docx]

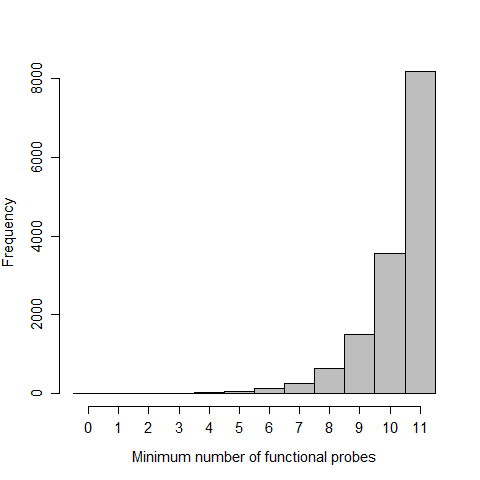


**Fig. S1** Distribution of positive probes per probe set. There is a maximum of 11 probes per probe set. 93.1% of designed probes showed expression in both subspecies of willow warblers (functional probe sets above 8). The average coverage was 10.24 functional probes per set.
