# Supplementary material for: Gene expression in the brain of a migratory songbird during breeding and migration
Source: Mov Ecol. 2016 Feb 15;4:4. doi: 10.1186/s40462-016-0069-6 (PMC4753645; doi:10.1186/s40462-016-0069-6)
Supplement: Additional file 1: — The distribution of positive probes per probe set is shown in Figure S1. The hierarchical clustering including the discarded samples is shown in Figure S2. Gene IDs, fold-change differences and FDR P-values for each of the 22,109 probe sets are available in Table S1. Microarray data are available in the ArrayExpress database (www.ebi.ac.uk/arrayexpress) under accession number E-MTAB-4102. (ZIP 1810 kb) [file 40462_2016_69_MOESM1_ESM.zip › Figure S2.docx]

Fig S2


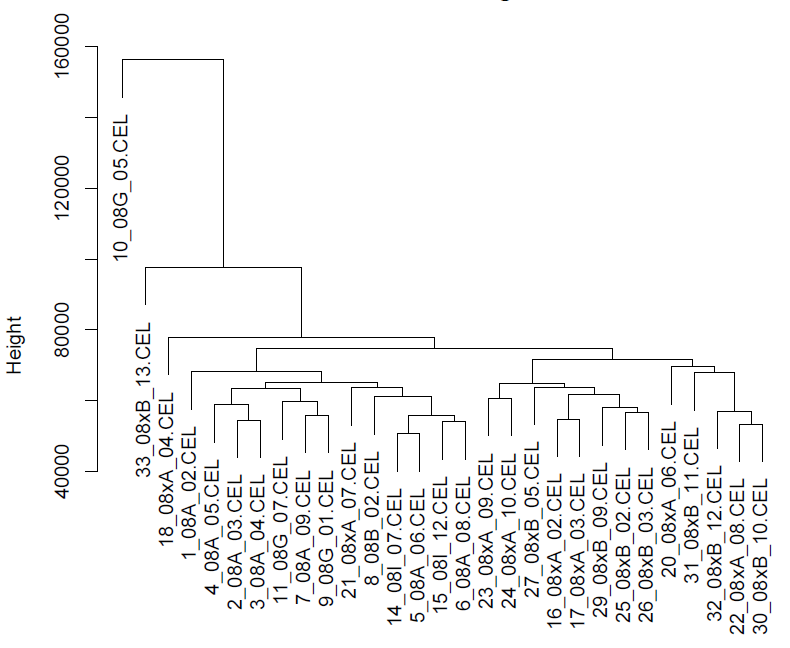


**Fig. S2** Hierarchical clustering of the array expression profiles for the 26 male willow warblers included in the analyses and the 3 samples excluded: 10_08G_05.CEL was a strong outlier, 33_08xB_13.CEL an adult male in the autumn sample and 18_08xA_04.CEL a female.
